# Potter Cove's Heavyweights: Estimation of Species' Interaction Strength of an Antarctic Food Web

**DOI:** 10.1002/ece3.70389

**Published:** 2024-11-03

**Authors:** Iara Diamela Rodriguez, Leonardo Ariel Saravia

**Affiliations:** ^1^ Instituto de Ciencias (ICI), Universidad Nacional de General Sarmiento (UNGS) Buenos Aires Argentina; ^2^ Centro Austral de Investigaciones Científicas (CADIC‐CONICET) Ushuaia Argentina

**Keywords:** Antarctic ecosystem, climate change, marine food web, species interaction strength

## Abstract

In the West Antarctic Peninsula, global warming has led to severe alterations in community composition, species distribution, and abundance over the last decades. Understanding the complex interplay between structure and stability of marine food webs is crucial for assessing ecosystem resilience, particularly in the context of ongoing environmental changes. In this study, we estimate the interaction strength within the Potter Cove (South Shetland Islands, Antarctica) food web to elucidate the roles of species in its structure and functioning. We use these estimates to calculate food web stability in response to perturbations, conducting sequential extinctions to quantify the importance of individual species based on changes in stability and food web fragmentation. We explore connections between interaction strength and key topological properties of the food web. Our findings reveal an asymmetric distribution of interaction strengths, with a prevalence of weak interactions and a few strong ones. Species exerting greater influence within the food web displayed higher degree and trophic similarity but occupied lower trophic levels and displayed lower omnivory levels (e.g., macroalgae and detritus). Extinction simulations revealed the key role of certain species, particularly amphipods and the black rockcod *Notothenia coriiceps*, as their removal led to significant changes in food web stability and network fragmentation. This study highlights the importance of considering species interaction strengths in assessing the stability of polar marine ecosystems. These insights have crucial implications for guiding monitoring and conservation strategies aimed at preserving the integrity of Antarctic marine ecosystems.

## Graphical abstract

This study investigates the Potter Cove food web in the West Antarctic Peninsula, assessing species interactions and ecosystem stability in the face of environmental changes. Through analysis of interaction strengths and extinction simulations, key species such as amphipods and the black rockcod *Notothenia coriiceps* are highlighted for their role in maintaining stability and mitigating network fragmentation, underscoring the importance of understanding species interactions for conservation efforts in Antarctica.

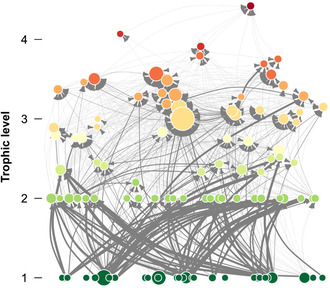

## 1 Introduction

The West Antarctic Peninsula has experienced the most intense warming on the planet in the last 50 years (Ducklow et al. 2013; Turner et al. 2014), with direct impacts on the cryosphere. As a result, the glacier in Potter Cove has been rapidly receding since 1950 (Rückamp et al. 2011), which has generated cascading effects in terms of freshwater input with sediment run‐off (Schloss et al. 2012), leading to profound changes on the benthic and pelagic communities (Sahade et al. 2015; Garcia et al. 2019; Braeckman et al. 2021; Deregibus et al. 2023).

Within an ecosystem, species are interconnected through feeding relationships, which shape energy flows and create complex food webs. One of the greatest challenges is to predict the effect of human activity on these complex webs of interactions among species. Species interactions mediate how changes in the physical and chemical environment play out throughout the ecosystem. Impacts affecting one species can have cascade effects on others, either directly or indirectly, depending on the pattern of strength of these connections. In the face of increasing mean global temperature caused by global climate change, understanding the effect of species on the stability of ecological communities is a pressing issue.

The exploration of food webs has significantly enhanced our comprehension of species' ecological roles and their impact on ecosystem functionality and resilience (Belgrano et al. 2005; Landi et al. 2018). Most food web studies have focused on binary representations, primarily examining species' presence or absence and their interactions (Dunne, Williams, and Martinez 2002; Kortsch et al. 2015; Olivier and Planque 2017; Marina, Salinas, et al. 2018). However, a deeper understanding recognizes that food webs possess inherent complexities in the form of weighted interactions, where the strengths of species interactions vary. Integrating weighted links based on interaction strengths in food web studies provides valuable ecological insights, especially when examining ecosystem function and stability. Understanding the pattern of these interaction strengths becomes pivotal in assessing and predicting food web stability.

Interaction strength in food webs estimates the magnitude of one species' effect on another and allows for differentiating the importance of species interaction. Several methodologies have been applied to estimate interaction strength in food webs that can require a great variety of empirical data, most of them using species biomass (Gauzens et al. 2019; Calizza et al. 2021; Gellner, McCann, and Hastings 2023). Here, we applied the method proposed by Pawar, Dell, and Savage (2012) that combines data on consumer and resource body masses, resource density, and consumer search space (interaction dimensionality) to obtain interaction strengths estimates for each pairwise predator–prey interaction. The novelty of this method is that it changes the coefficients that relate body size to metabolism according to whether the species moves in 2D or 3D, and it has the advantage that resource density and species biomass or density are not mandatory.

While the Potter Cove food web topology, complexity, and stability have been largely studied (Marina, Salinas, et al. 2018; Marina, Saravia, et al. 2018; Cordone et al. 2018, 2020; Rodriguez et al. 2022), this study aims to go beyond a purely topological (presence/absence) assessment of who eats whom in the Potter Cove ecosystem. Our goal is to analyze the trophic network structure quantitatively and to evaluate the species' role in the food web structure and stability, considering the strength of interactions. To achieve this, in the first place, we estimated the strength of each pairwise interaction. Secondly, we characterized species' roles considering both weighted and unweighted properties and evaluated the relationship between these metrics. Finally, we assessed the impact of individual species on food web stability and fragmentation through simulations of sequential extinctions.

## 2 Materials and Methods

### 2.1 Description of the Study Area

**FIGURE 1:**
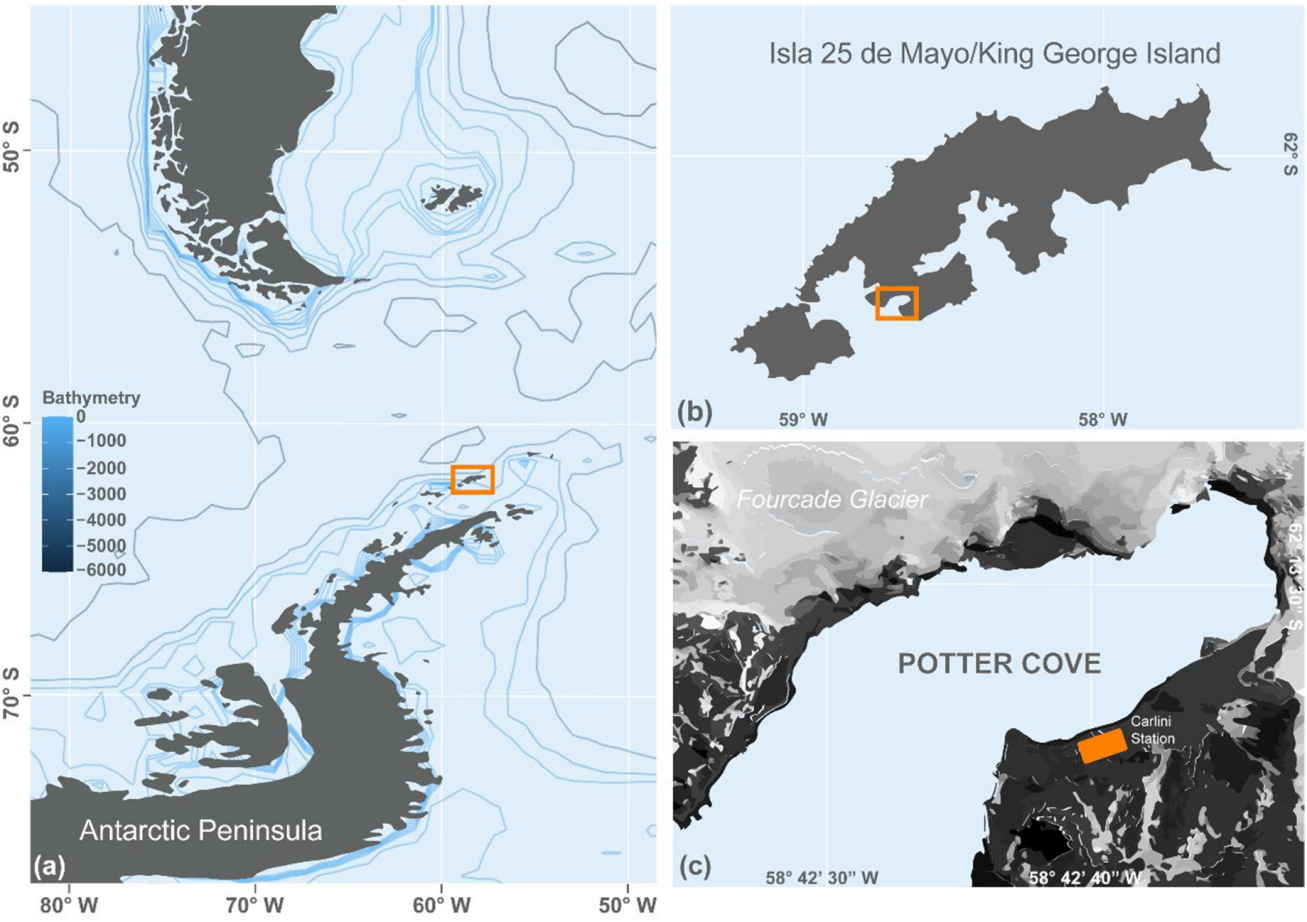
Map of Potter Cove and its location at Isla 25 de Mayo/King George Island (South Shetland Islands, Antarctic Peninsula). The bicontinental map (a) was drawn using the “marmap” R package (Pante and Simon‐Bouhet 2013). Contour shape file for Isla 25 de Mayo/King George Island (b) was obtained from www.ign.gob.ar, and Potter Cove's (c) from Neder et al. (2022).

Potter Cove (62° 14' S, 58° 38' W) is an ~9 km^2^ fjord located at Isla 25 de Mayo/King George Island, South Shetland Islands, on the West Antarctic Peninsula (Figure 1). Potter Cove's high‐latitude location results in fluctuating environmental conditions driven by the strong seasonality in the photoperiod length. The winter reduction in irradiance and temperature regulates several environmental variables, including incident radiation, sea‐ice extent, mixing layer depth, water column particulate matter, and nutrient concentration.

### 2.2 Potter Cove Food Web Dataset

We used a well‐resolved food web that documents 649 feeding links between 110 species that inhabit Potter Cove (Rodriguez et al. 2022). The species diet information was collected and compiled from gut content studies and personal communication with experts. The Potter Cove food web can be considered representative of the summer season since data were collected during austral summer months when most research campaigns are carried out. More detailed information on Potter Cove food web assembly can be found in Marina, Salinas, et al. (2018) and Rodriguez et al. (2022).

### 2.3 Interaction Strength Estimation

We estimated the strength of each pairwise interaction in the food web following Pawar, Dell, and Savage (2012) methodology, considering consumer (predator) and resource (prey) body mass and the interaction dimensionality (ID). First, we compiled information about resources and consumers' body mass $m_{R}$ and $m_{C}$. Then, the ID was classified as two or three dimensions based on the species movement space and habitat. We assign 2D when both predator and prey move in 2D (e.g., both are benthic) or if a predator moves in 3D and a prey in 2D (e.g., pelagic predator on benthic prey). The ID was classified as 3D when both predator and prey move in 3D (e.g., both pelagic) or if the predator moves in 2D and the prey in 3D (e.g., benthic predator, pelagic prey) (Pawar, Dell, and Savage 2012).

The main equation we used to estimate the interaction strength (IS) was:

$$\text{IS}=\frac{\alpha \;x_{R}\;m_{R}}{m_{C}}$$

where α is the search rate, $\;x_{R}$ is the resource density, and $m_{R}$ and $m_{C}$ are the body mass of the resource and the consumer, respectively (Pawar, Dell, and Savage 2012).

We obtained estimates for the search rate (α) from the power‐law scaling relationship with the consumer mass, determined by ordinary least squares regression, but with different coefficients for both dimensional search space (Pawar, Dell, and Savage 2012). When available, we used empirical resource density ($\;x_{R}$) acquired from bibliography, otherwise, we estimated it from the scaling relationship with the resource body mass, since it scales as power‐law with different exponents in 2D and in 3D (Pawar, Dell, and Savage 2012). For resources such as macroalgae, sponges, necromass, and fresh and aged detritus, where body mass and/or density are independent from the consumer, a value of 1 was assigned $m_{R}$ and $m_{C}$. Consequently, the interaction strength was solely dependent on consumer biomass (Pawar, Dell, and Savage 2012). The equations for estimating the search rate and the resource density are specified in the Supporting Information.

Since the exponents reported by Pawar, Dell, and Savage (2012) have associated standard deviations from the estimation through linear regressions, we used these uncertainties to measure the variability in interaction strength estimates. We generated 1000 random samples of the exponents, assuming a normal distribution with a mean based on the estimated exponent and a standard deviation equal to the reported standard error. Then, we calculated interaction strength values for each sample, leading to distributions of interaction strength estimates for each pairwise interaction. Since these interaction strength distributions showed right‐skewed tendencies, we used the median IS to describe the central tendency.

We fitted the interaction strength distribution (i.e., medians for each interaction) to various models including exponential, gamma, log‐normal, normal, power‐law, and uniform using maximum likelihood (McCallum 1999) and chose the best model using the Akaike Information Criterion (Burnham and Anderson 2004).

### 2.4 Species Properties

To characterize the species' role in food web structure and stability, we calculated unweighted food web properties. Unweighted properties are topology‐based metrics applied to binary food webs that only describe species presence/absence, where all trophic links are considered equally important in the food web. We calculated four commonly used topological species properties in food web studies: (a) trophic level, (b) degree, (c) omnivory, and (d) trophic similarity. Trophic level represents the number of feeding links separating a species from the base of production in a food web. Top predators and primary producers are expected to have large effects on their communities through top‐down and bottom‐up control (Cirtwill et al. 2018). The degree of a species is calculated as the sum of all in‐ (number of prey) and out‐ (number of predators) trophic interactions. Species with many connections tend to have a large impact on food structure, functioning, and stability, because perturbations affecting them can have a cascading effect, impacting many other species within the ecosystem (Cirtwill et al. 2018). Omnivory is a feeding strategy in which a consumer feeds at multiple trophic levels. Omnivore species can adapt faster to variation in prey abundances, and it gives trophic flexibility to an ecosystem by presenting alternative energy pathways in the face of perturbations (Wootton 2017). Trophic similarity is an index that measures the degree of overlap in the feeding relationships between species, considering both their roles as consumers and as resources within a food web. It reflects how similar the trophic niches of different species are, indicating how much they rely on similar types of prey or are preyed upon by similar predators (Morlon, Kefi, and Martinez 2014; Delmas et al. 2019). Formulas used to obtain the above species' properties are described in Supporting Information.

To study the relationship between species topological properties and interaction strength, we performed quantile regression at quantile 0.25, 0.5 (the median), and 0.75 between the log total interaction strength, representing the sum of the interaction strength for all interactions (both in and out) involving a given species, and each of the species topological properties. Slope significance of the quantile regressions was assessed using the bootstrap method (Koenker 2005).

### 2.5 Species Impact on Food Web Stability and Fragmentation

To analyze the individual impact of species on food web stability, we performed species removal simulations, sequentially deleting the first 50 species in decreasing order of total interaction strength, trophic level, degree, omnivory, trophic similarity, and intermodule connectivity. This last metric is associated with food webs' tendency to be organized into modular patterns, where groups of species interact more strongly with each other than with species from other groups. Species can assume various roles within this modular organization based on the distribution of trophic links within their own module and/or across modules. The intermodule connectivity estimates the distribution of interaction of a species across modules.

After each species extinction, we examined the impact on food web stability and fragmentation. We did not analyze secondary extinctions after the removal of a species.

Stability is a multidimensional concept that generally refers to an ecosystem's ability to maintain its state over time, despite external and internal forces that may disrupt it. A well‐studied aspect of stability is local stability, which evaluates how a system responds to small disturbances near an equilibrium point. Local stability is assessed using the Jacobian matrix, which is constructed from the partial derivatives of the system's dynamics. These dynamics are represented by the adjacency matrix (*A*), where each element *a*_*ij*_ equals 1 if species *j* preys on species *i*, and 0 otherwise. The system's stability is determined by evaluating the Jacobian matrix at equilibrium points, the eigenvalue.

In this study, we used the Quasi Sign−Stability (QSS) metric (Allesina and Pascual 2008) to evaluate stability. This metric assesses stability based on the pattern of signs in the Jacobian matrix, which indicates the types of interactions (negative for resources, positive for consumers), while randomizing the magnitudes of these interactions (interaction strength), with the maximum value set to the estimated interaction strength (Borrelli and Ginzburg 2014; Allesina and Pascual 2008; Grilli, Rogers, and Allesina 2016; Saravia et al. 2022). Then, stability was measured as the average of the real part of the maximum eigenvalue, which describes the rate at which a small perturbation decays or amplifies over time near an equilibrium point. A more negative index indicates a more stable food web with a reduced probability of perturbation amplification.

In predator–prey networks, system stability can be achieved by reducing the number of interacting species, decreasing connectivity between species, or increasing species self‐regulation (the direct effects of species on themselves) (Allesina and Tang 2012; Barabás, Michalska‐Smith, and Allesina 2017). In the Jacobian matrix, self‐regulatory effects are represented as negative entries along the diagonal. However, in our analysis, these self‐regulation terms were set to zero due to a lack of empirical data for all species, resulting in maximum eigenvalues that are predominantly positive, indicating potential system instability. The maximum eigenvalue, thus, reflects the degree of species self‐regulation required for the food web to achieve stability (Grilli, Rogers, and Allesina 2016). Species whose removal significantly alters the maximum eigenvalue, and thus system stability, are identified as key species within the network. If the removal of a species leads to a sharp increase in the maximum eigenvalue, suggesting increased instability, it indicates that the species likely required substantial self‐regulation for the network to remain stable.

In modular food webs, typically, a few key species, with high connectivity both between and within modules, play a crucial role in linking the entire food web. We measured the cohesion of the food web by calculating the number of connected components after the removal of a species. These connected components represent species or subgroups unconnected to others and can be considered an extreme form of modules. The number of components in ecological networks is important for the overall structure and resilience of the ecosystem. When an ecological network becomes separated into smaller components, it represents distinct channels of energy flow and species interactions. This characteristic could confer an advantage in scenarios where the network is subjected to perturbations, as it prevents the effects of perturbations from propagating to other components (Stouffer and Bascompte 2011; Gilarranz et al. 2017). However, a higher number of components can be detrimental to the network. It can lead to fragmented energy pathways, reduced energy transfer, and limited species interactions. We considered the food web fragmented when there was more than one component, with the species responsible for the fragmentation considered a key species contributing to maintaining a cohesive food web structure.

We conducted 1000 simulations for the removal of each species, calculating the maximum eigenvalue for the food web in each case. We plotted the sequential species' extinction results, according to the different species properties, and their effect on food web stability and fragmentation.

### 2.6 Data Analysis and Availability

All analyses, simulations, and graphs were performed in R version 4.3.1 (R Core Team 2024) using the R packages “igraph” (Csardi and Nepusz 2006), cheddar (Hudson et al. 2013), and the “multiweb” R package to calculate all network metrics and food web simulations (Saravia 2024).

## 3 Results

### 3.1 Interaction Strength Distribution

**FIGURE 2:**
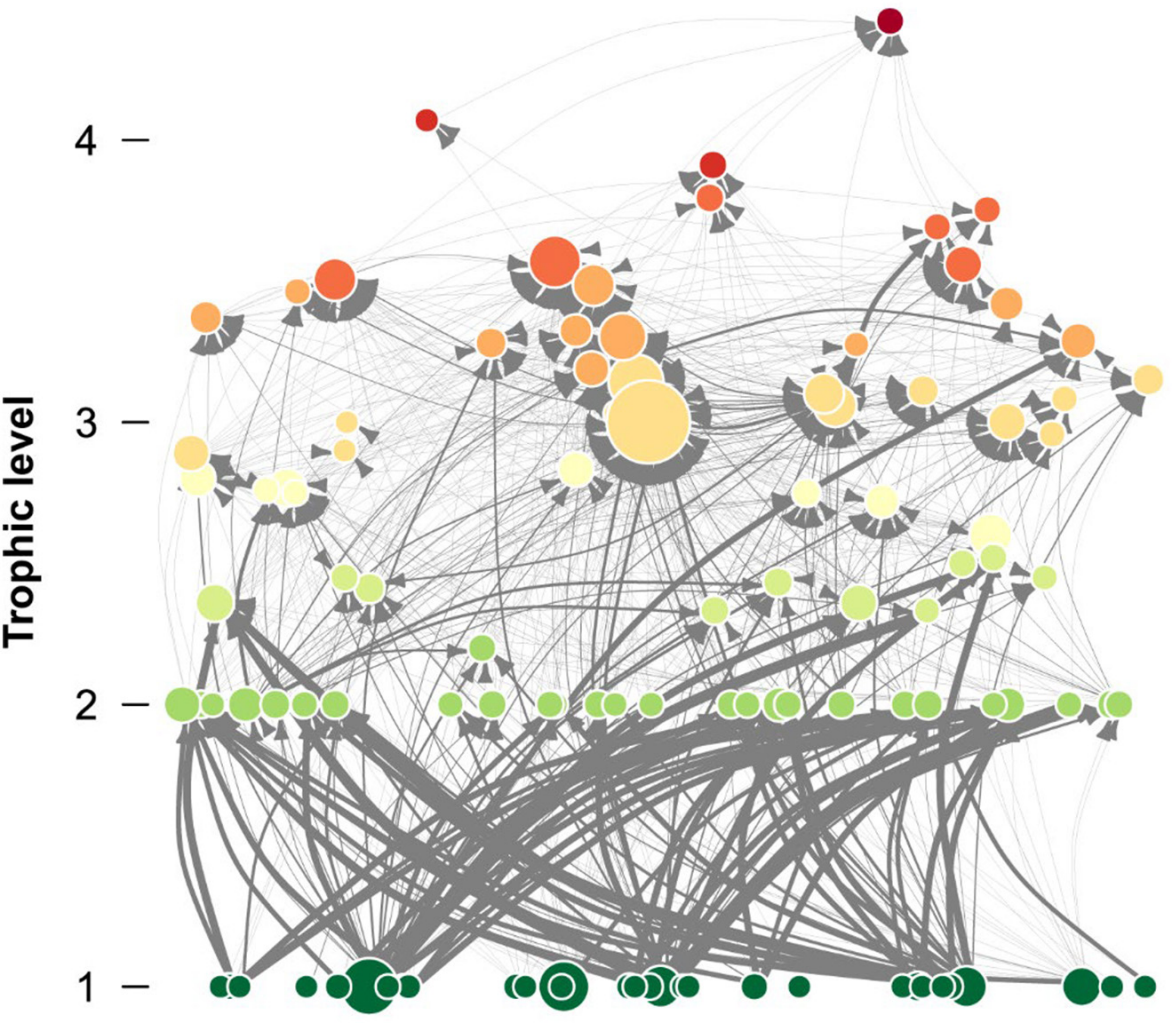
Graphic representation of the Potter Cove food web. Species (nodes) are arranged vertically and colored by trophic level. The size of the nodes indicates the total number of interactions (degree). Predator–prey interactions are represented by the arrows, from prey to predator, and arrow thickness is proportional to the strength of the interaction.

**FIGURE 3:**
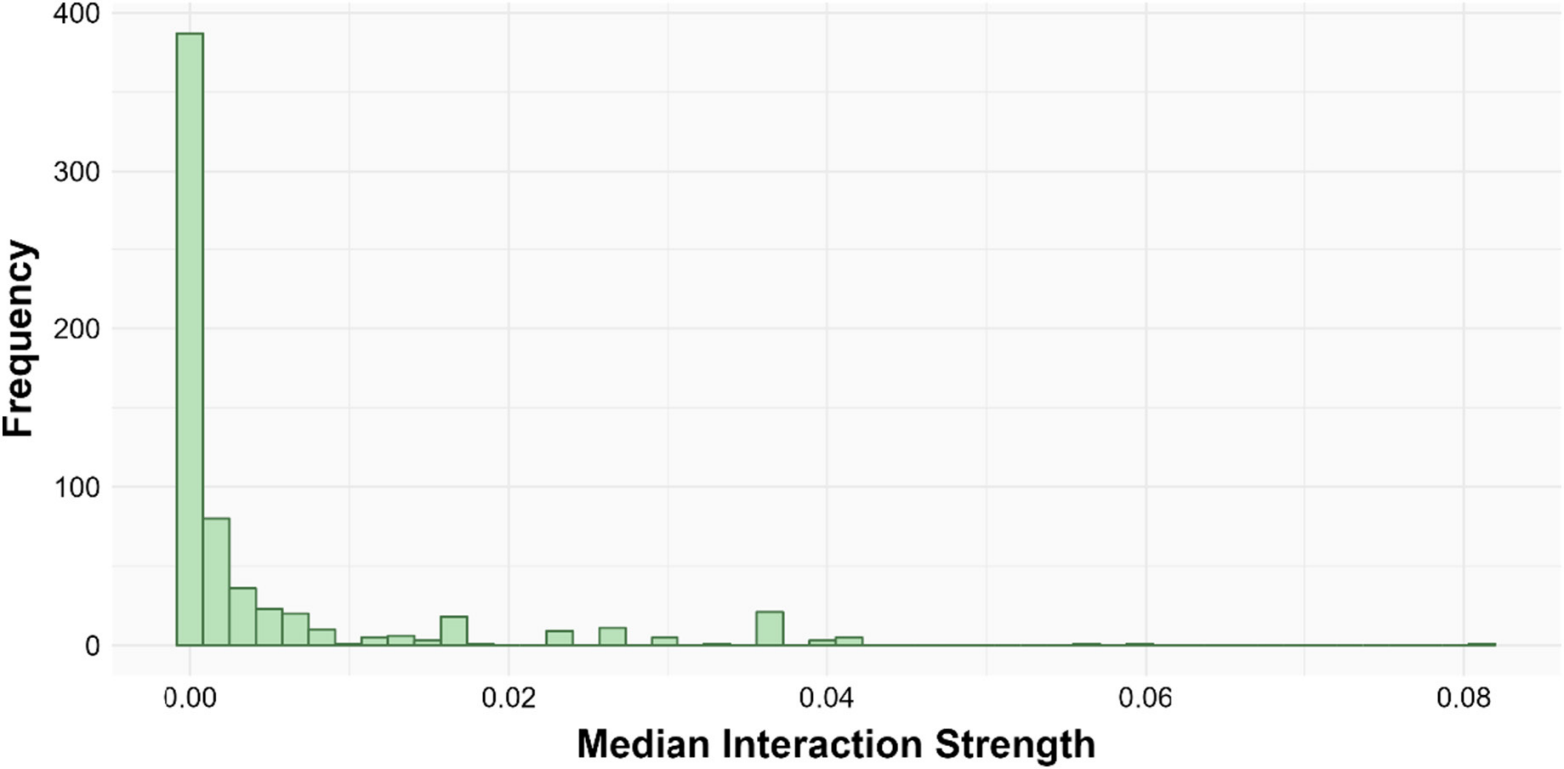
Frequency distribution of the median interaction strengths for the Potter Cove food web. Total number of interactions = 649. The distribution was best fitted to a gamma model.

The interaction strength distribution analysis of the Potter Cove food web showed that the gamma model was the best fit, according to the AIC analysis, with a high proportion of weak interactions, and only a few strong interactions (Figures 2 and 3, Supporting Information Table S1).

### 3.2 Species Interaction Strength and Topological Properties

**FIGURE 4:**
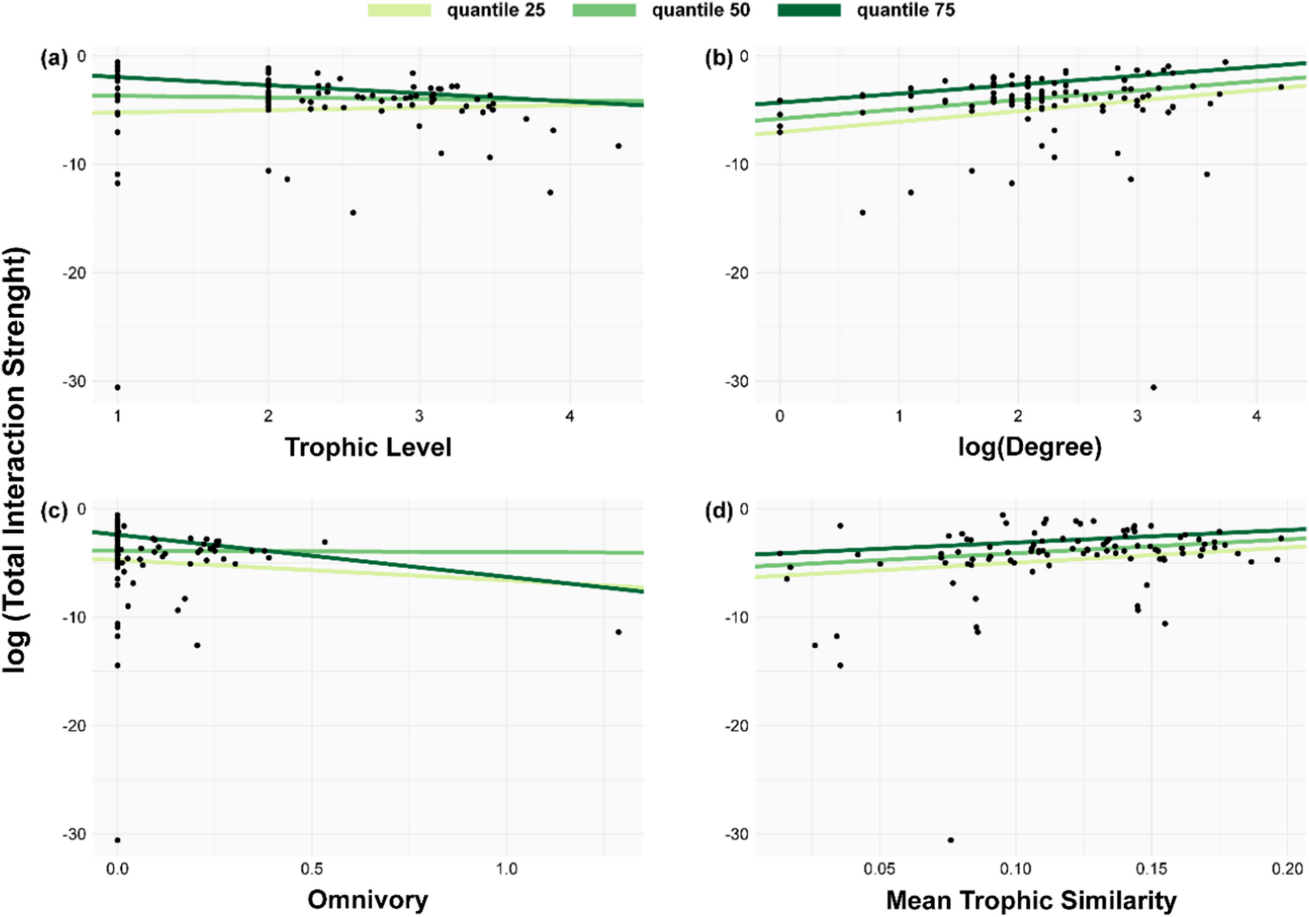
Relationships between weighted (total interaction strength) and unweighted food web properties. We fitted quantile regressions (light green line = quantile 25, medium green line = quantile 50, dark green line = quantile 75) to show the tendency between log total interaction strength and (a) trophic level, (b) degree, (c) omnivory, and (d) trophic similarity.

We found that species' total interaction strength was positively associated with both degree and trophic similarity in all three quantile regressions (Figure 4b,d, Supporting Information Table S2). The species trophic level and omnivory showed a negative relationship with the total interaction strength for the quantile 75 regression (Figure 4a,c, Supporting Information Table S2). However, no significant relationship was observed for regressions at quantiles 25 and 50 for both unweighted species properties (Supporting Information Table S2).

This suggests that species exhibiting the highest interaction strength tend to exhibit a higher degree and higher dietary and predator overlap, occupy lower trophic positions, and display lower levels of omnivory. The identity of species that exert the most substantial influence on Potter Cove food web are basal species (detritus and some species of macroalgae) and grazers (mostly amphipods) (Supporting Information Table S2a).

### 3.3 Species Impact on Food Web Stability and Fragmentation

**FIGURE 5:**
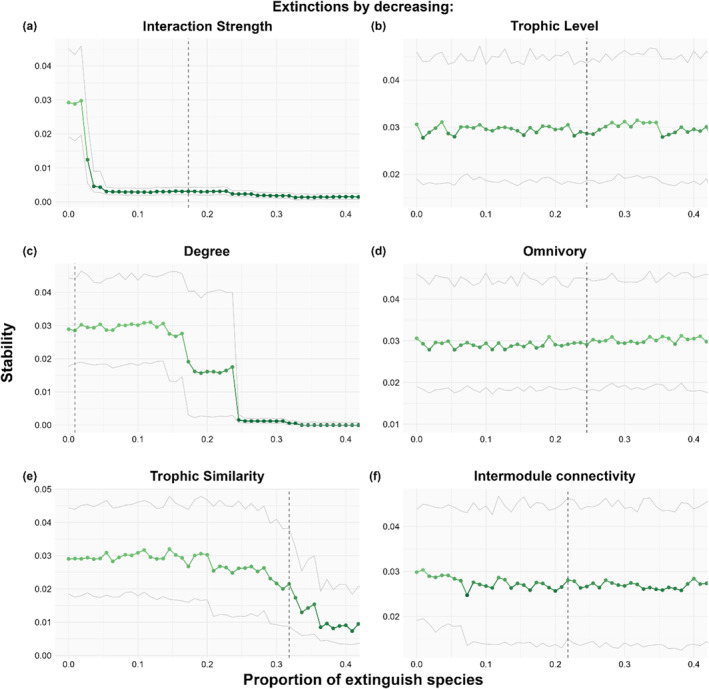
Effects on stability (median maximum eigenvalue) when removing species sequentially based on decreasing weighted and unweighted network properties: (a) interaction strength, (b) trophic level, (c) degree, (d) omnivory, (e) trophic similarity, and (f) intermodule connectivity. Gray continuous lines represent interquartile stability values. Dark gray dotted vertical line represents the species whose extinction results in the fragmentation of the food web into more than one compartment.

The extinction analyses revealed that removal performed by different species properties has distinct effects on food web stability (Figure 5). While no clear pattern emerged in stability when removing species by decreasing trophic level, omnivory, and intermodule connectivity (Figure 5b,d,f), we observed that network stability increased after the removal of species with higher interaction strength, degree, and trophic similarity (Figure 5a,c,e).

When extinctions were performed by decreasing interaction strength, we found that the removal of the amphipods *Prostebbingia sp*. and *P. gracilis*, the third and fourth species with higher interaction strength, substantially increased food web stability (Figure 5a, Supporting Information Table S3a). In sequential removals of high‐degree species, the amphipods *Gondogeneia antarctica* and *Prostebbingia gracilis* caused a major increase in food web stability (Figure 5c, Supporting Information Table S3c).

Regarding food web fragmentation, we observed that the removal of the fish *Notothenia coriiceps*, in extinctions by trophic level, degree, omnivory, and intermodule connectivity (Figure 5b–d,f, Supporting Information Table S3b–d,f), was responsible for the fragmentation of the food web into nine compartments. For extinctions performed by interaction strength, the amphipod *Paradexamine fissicauda* caused the fragmentation of the food web into two compartments, which remained unchanged until *N. coriiceps* was removed, dividing the food web into 14 compartments (Figure 5a, Supporting Information Table S3a). In the simulations run by decreasing trophic similarity, *N. coriiceps* did not contribute to the fragmentation of the food web. Instead, *Nacella concinna* was responsible for the fragmentation of the network (Figure 5e, Supporting Information Table S3e). Notably, network fragmentation does not seem related to stability as fragmentation points do not align with significant stability changes.

## 4 Discussion

### 4.1 Interaction Strength Distribution

The estimation of the species interaction strength for the Potter Cove food web allowed us a better understanding of species' role in food web stability. We found that the distribution of interaction strength was skewed toward a few strong and many weak links, as observed in extensive theoretical and empirical studies (Drossel, McKane, and Quince 2004; Wootton and Emmerson 2005; Kortsch et al. 2021; Marina, Saravia, and Kortsch 2024). This asymmetrical pattern has been proposed to promote ecosystem persistence and stability (McCann, Hastings, and Huxel 1998; Drossel, McKane, and Quince 2004; Emmerson and Yearsley 2004; Bascompte, Melián, and Sala 2005).

### 4.2 Species Interaction Strength and Topological Properties

We employed a range of descriptors, including unweighted and weighted metrics, to elucidate what makes a species important in the Potter Cove food web. Our findings revealed a positive correlation between a species' interaction strength and its degree, as well as trophic similarity. Conversely, trophic level and omnivory exhibited a negative correlation with the highest levels of interaction strength. The species that exert the most substantial influence on Potter Cove food web are basal species (detritus and some species of macroalgae) and grazers (mostly amphipods), with a high number of interactions and trophic redundancy. This theoretical framework aligns with empirical evidence that the large biomass macroalgae dominating shallow benthic communities, along with the detritus derived from them, play a fundamental role as the energetic base of the Potter Cove food web (Gómez and Huovinen 2020) and support a high‐density assemblage of invertebrates, especially amphipods (Huang et al. 2007). While macroalgae have a great influence in shaping the structure of the Potter Cove food web, their direct impact on its stability appears to be less important. Local losses of macroalgae species do not immediately destabilize the food web; rather, they exhibit relative robustness until a high critical stress threshold is surpassed. Beyond this point, negative effects propagate rapidly throughout the entire food web, leading to its collapse (Cordone et al. 2018, 2020).

### 4.3 Species Impact on Food Web Stability and Fragmentation

The Potter Cove food web tends to be more stable and less connected upon the removal of species, as expected. Our study underscores that species exhibiting high total interaction strength, degree, and trophic similarity need to be considered with particular attention when trying to predict the effects of perturbations on the Potter Cove ecosystem. The extinction simulations reveal a threshold behavior in stability—meaning it does not increase gradually—when species are removed by interaction strength, degree, and to a lesser extent by trophic similarity. This is significant as it suggests nonlinear effects and confirms the existence of key species that produce these thresholds. This pattern is not observed with omnivory, trophic level, or intermodule connectivity. Contrary to expectations, species with the highest degree or interaction strength are not necessarily the most important. Instead, our analysis suggests that species interaction strength and degree have a strong effect on the functioning and stability of the Potter Cove food web.

Fragmentation is an extreme form of compartmentalization, and compartmentalized food webs are generally associated with increased stability. However, in our study, stability appears to be unrelated to the fragmentation of the network, as extinctions causing fragmentation do not correspond to shifts in stability. Although the extinction of individual species did not directly affect network stability, increased compartmentalization might make the network more vulnerable to further species losses within isolated modules. This suggests that while fragmentation alters the structure of the network, (1) the stability metric used in this study may not fully capture the resulting dynamics, and (2) the relationship between fragmentation and stability may not be so straightforward. D'Alelio et al. (2019) also observed that modular reorganization under environmental changes can occur without affecting stability. Future analyses should explore food web compartmentalization with an emphasis on species interaction strength by investigating weighted modularity, which may offer a more nuanced understanding of how species roles influence network stability in the context of extinctions.

Our results show that fragmentation is primary linked to the species degree, rather than intermodule connectivity. In the modular configuration of food webs, species causing fragmentation are not only responsible for connecting different modules (high intermodule connectivity) but also need to have a great number of interactions (high degree) (Guimerà and Amaral 2005). If these connecting species—also known as network connectors—go extinct, entire modules can become disconnected. This is the case of the black rockcod, *Notothenia coriiceps*. A generalist, omnivorous, top predator fish with the highest degree, consistently contributes to the fragmentation of the Potter Cove food web in most extinction simulations (by decreasing interaction strength, trophic level, degree, omnivory, and intermodule connectivity). These results further support its potential status as a keystone species in this ecosystem. Previous research examining topological and modular characteristics of the Potter Cove food web has highlighted the central role of *N. coriiceps* in enhancing overall network connectivity (Marina, Salinas, et al. 2018; Rodriguez et al. 2022). Salinas et al. (2024) observed that the removal of *N. coriiceps* resulted in cascading effects on metrics such as connectance, modularity, and stability.

Furthermore, our different analysis consistently points at grazers, like the limpet *Nacella concinna* and the amphipods *Paradexamine fissicauda*, *Gondogeneia antarctica*, and species of the genus *Prostebbingia*, as another group of key species of the Potter Cove food web. These grazers are particularly important because they present high interaction strengths and *N. concinna* and *P. fissicaudata* when removed, lead to the fragmentation of the network. Amphipods constitute an important and abundant component of Antarctic benthic communities and, alongside macroalgae, represent the primary food sources for Antarctic fish, such as *N. coriiceps* (Barrera‐Oro et al. 2019).

In this study, we calculated interaction strengths by directly incorporating empirical data on species' body mass and density—key factors for accurately capturing the patterns of community interaction strength structure (Berlow et al. 2004, 2009; Pawar, Dell, and Savage 2012). The inclusion of species density allows for a more realistic representation of the actual interactions occurring in the Potter Cove ecosystem, as it influences the impact one species can have on another. However, we recognize the limitations of this approach, especially when considering sequential extinctions based on interaction strengths. As species are removed, subsequent shifts in biomass and population densities can alter interaction strengths, potentially leading to different outcomes in the food web's stability and structure. While this method provides an ecologically grounded analysis, it is important to recognize the inherent limitations in interpreting the results. Future studies could explore complementary approaches that either account for these shifts dynamically or use biomass‐independent metrics to provide a more nuanced understanding of interaction strengths under different extinction scenarios.

### 4.4 Comparison With Another Antarctic Food Web

Our findings show some discrepancies with those of Marina, Saravia, and Kortsch (2024), who applied the same method to calculate interaction strength for the Weddell Sea (Antarctica) food web. Both studies consistently show a relationship between interaction strength and degree, suggesting that species with more interactions tend to exert stronger impacts on the food web. However, while Marina and colleagues found a strong positive correlation between interaction strength and trophic level, our study reveals a negative correlation. This discrepancy may stem from the profound structural differences between the ecosystems studied.

The Weddell Sea food web encompasses a vast area of 450 km^2^ and includes 490 species and 16,041 predator–prey interactions—almost five times more species and 24 times more interactions than in Potter Cove. The Weddell Sea also includes a considerable higher number of predators and land‐based species, absent in Potter Cove, which may explain the inverse relationship between interaction strength and trophic level observed in our study.

In the Weddell Sea, species with lower interaction strengths are predominantly benthic, while those with higher interaction strengths occupy intermediate to top positions in the food web and are found in pelagic and benthopelagic habitats. In contrast, in Potter Cove, species with high interaction strengths are primarily benthic and include basal organisms and herbivores. This difference likely reflects the varying reliance on primary production in these ecosystems. The Weddell Sea, with water depths ranging from 200 to 500 m, supports species that rely less on benthic primary production, with many organisms, including pelagic, benthopelagic, and land‐based species, depending on pelagic primary production from phytoplankton. In contrast, in Potter Cove, macroalgae contribute significantly to biomass and serve as a major energy source for the benthic community. These findings highlight how different sources of primary production, by shaping energy channels, influence the patterns of interaction strengths within communities, which form the basis of ecosystem structure, function, and stability (De Ruiter, Neutel, and Moore 1995).

Despite the great differences between both Antarctic ecosystems and food web structure, Marina, Saravia, and Kortsch (2024) also found, as we did, that only a few species significantly impact food web stability when removed, and that those species were characterized by high interaction strength and a large number of interactions (degree).

The discrepancies between their results and those of our study highlight the inadequacy of relying solely on unweighted (topological) indices as reliable indicators of interaction strength. Conversely, utilizing interaction strength estimations applied to the study of food web stability appears to be a valuable tool for identifying key species within ecosystems, beyond the unique characteristics and structure of individual food webs.

### 4.5 Implications of the Study in a Changing World

Climate change–induced warming in Potter Cove is substantially changing the community composition, species distribution, and abundance. This warming has led to glacier retreat, creating new habitats for macroalgal colonization, and increased glacier sediment runoff, impacting the photosynthetic rates of primary producers and intensifying competition among species (Deregibus et al. 2016). Simultaneously, Barrera‐Oro et al. (2019) observed changes in the feeding selectivity of *N. coriiceps* on amphipods, correlating with shifts in the macroalgae‐associated amphipod community. These shifts are linked to alterations in salinity and changes in water column mixing processes, which regulate phytoplankton biomass accumulation (Schloss, Ferreyra, and Ruiz‐Pino 2002; Schloss et al. 2012). However, the net effects of climate change on macroalgae and other key species, such as amphipods and fish, remain uncertain and represent a challenge to elucidate.

The methodology applied in this study shows great potential for guiding monitoring and conservation strategies, focused on key species, aimed at protecting the integrity of Antarctic marine ecosystems in times of rapid climate changes. Through the incorporation of species interaction strength into our analysis of the Potter Cove food web, we have identified characteristics and potential key species that exert significant influence over both the structure and stability of the ecosystem. The nonlinear effects observed in the stability analysis stress the importance of protecting these key species to maintain ecosystem resilience.

## Author Contributions

**Iara Diamela Rodriguez:** conceptualization (equal), data curation (lead), formal analysis (equal), funding acquisition (equal), investigation (equal), methodology (equal), software (supporting), validation (equal), visualization (lead), writing – original draft (lead), writing – review and editing (lead). **Leonardo Ariel Saravia:** conceptualization (equal), formal analysis (equal), funding acquisition (equal), investigation (equal), methodology (equal), project administration (lead), software (lead), supervision (lead), validation (equal), visualization (supporting), writing – original draft (supporting), writing – review and editing (supporting).

## Conflicts of Interest

The authors declare no conflicts of interest.

### Open Research Badges

This article has earned an Open Data badge for making publicly available the digitally‐shareable data necessary to reproduce the reported results. The data is available at https://github.com/123iamela/pottercove‐IS and https://doi.org/10.5281/zenodo.10790590.

## Supporting information


Data S1.

## Data Availability

All source code and data that support the findings of this study are openly available in GitHub at https://github.com/123iamela/pottercove‐IS and Zenodo at https://doi.org/10.5281/zenodo.10790590.

## References

[ece370389-bib-0001] Allesina, S. , and M. Pascual . 2008. “Network Structure, Predator–Prey Modules, and Stability in Large Food Webs.” Theoretical Ecology 1, no. 1: 55–64. 10.1007/s12080-007-0007-8.

[ece370389-bib-0002] Allesina, S. , and S. Tang . 2012. “Stability Criteria for Complex Ecosystems.” Nature 483, no. 7388: 205–208. 10.1038/nature10832. 22343894

[ece370389-bib-0003] Barabás, G. , M. J. Michalska‐Smith , and S. Allesina . 2017. “Self‐Regulation and the Stability of Large Ecological Networks.” Nature Ecology & Evolution 1, no. 12: 1870–1875. 10.1038/s41559-017-0357-6. 29062124

[ece370389-bib-0004] Barrera‐Oro, E. , E. Moreira , M. A. Seefeldt , M. Valli Francione , and M. L. Quartino . 2019. “The Importance of Macroalgae and Associated Amphipods in the Selective Benthic Feeding of Sister Rockcod Species *Notothenia rossii* and *N*. *coriiceps* (Nototheniidae) in West Antarctica.” Polar Biology 42, no. 2: 317–334. 10.1007/s00300-018-2424-0.

[ece370389-bib-0005] Bascompte, J. , C. J. Melián , and E. Sala . 2005. “Interaction Strength Combinations and the Overfishing of a Marine Food Web.” Proceedings of the National Academy of Sciences 102, no. 15: 5443–5447. 10.1073/pnas.0501562102. PMC556268 15802468

[ece370389-bib-0006] Belgrano, A. , U. M. Scharler , J. Dunne , and R. E. Ulanowicz . 2005. Aquatic Food Webs: An Ecosystem Approach. Oxford: Oxford University Press. 10.1093/acprof:oso/9780198564836.001.0001.

[ece370389-bib-0007] Berlow, E. L. , J. A. Dunne , N. D. Martinez , P. B. Stark , R. J. Williams , and U. Brose . 2009. “Simple Prediction of Interaction Strengths in Complex Food Webs.” Proceedings of the National Academy of Sciences 106, no. 1: 187–191. 10.1073/pnas.0806823106. PMC2629248 19114659

[ece370389-bib-0008] Berlow, E. L. , A. Neutel , J. E. Cohen , et al. 2004. “Interaction Strengths in Food Webs: Issues and Opportunities.” Journal of Animal Ecology 73, no. 3: 585–598. 10.1111/j.0021-8790.2004.00833.x.

[ece370389-bib-0009] Borrelli, J. J. , and L. R. Ginzburg . 2014. “Why There Are So Few Trophic Levels: Selection Against Instability Explains the Pattern.” Food Webs 1, no. 1–4: 10–17. 10.1016/j.fooweb.2014.11.002.

[ece370389-bib-0010] Braeckman, U. , F. Pasotti , R. Hoffmann , et al. 2021. “Glacial Melt Disturbance Shifts Community Metabolism of an Antarctic Seafloor Ecosystem From Net Autotrophy to Heterotrophy.” Communications Biology 4, no. 1: 148. 10.1038/s42003-021-01673-6. 33514890 PMC7846736

[ece370389-bib-0011] Burnham, K. P. , and D. R. Anderson , eds. 2004. “Statistical Theory and Numerical Results.” In En Model Selection and Multimodel Inference, 352–436. New York: Springer. 10.1007/978-0-387-22456-5_7.

[ece370389-bib-0012] Calizza, E. , L. Rossi , G. Careddu , S. Sporta Caputi , and M. L. Costantini . 2021. “A Novel Approach to Quantifying Trophic Interaction Strengths and Impact of Invasive Species in Food Webs.” Biological Invasions 23, no. 7: 2093–2107. 10.1007/s10530-021-02490-y.

[ece370389-bib-0013] Cirtwill, A. R. , G. V. Dalla Riva , M. P. Gaiarsa , et al. 2018. “A Review of Species Role Concepts in Food Webs.” Food Webs 16: e00093. 10.1016/j.fooweb.2018.e00093.

[ece370389-bib-0014] Cordone, G. , T. I. Marina , V. Salinas , S. R. Doyle , L. A. Saravia , and F. R. Momo . 2018. “Effects of Macroalgae Loss in an Antarctic Marine Food Web: Applying Extinction Thresholds to Food Web Studies.” PeerJ 6: e5531. 10.7717/peerj.5531. 30225167 PMC6139014

[ece370389-bib-0015] Cordone, G. , V. Salinas , T. I. Marina , et al. 2020. “Green vs Brown Food Web: Effects of Habitat Type on Multidimensional Stability Proxies for a Highly‐Resolved Antarctic Food Web.” Food Webs 25: e00166. 10.1016/j.fooweb.2020.e00166.

[ece370389-bib-0016] Csardi, G. , and T. Nepusz . 2006. “The igraph Software Package for Complex Network Research.” InterJournal, Complex Systems, 1695. https://igraph.org.

[ece370389-bib-0017] D'Alelio, D. , B. Hay Mele , S. Libralato , M. Ribera d'Alcalà , and F. Jordán . 2019. “Rewiring and Indirect Effects Underpin Modularity Reshuffling in a Marine Food Web Under Environmental Shifts.” Ecology and Evolution 9, no. 20: 11631–11646. 10.1002/ece3.5641. 31695874 PMC6822054

[ece370389-bib-0018] De Ruiter, P. C. , A.‐M. Neutel , and J. C. Moore . 1995. “Energetics, Patterns of Interaction Strengths, and Stability in Real Ecosystems.” Science 269, no. 5228: 1257–1260. 10.1126/science.269.5228.1257. 17732112

[ece370389-bib-0019] Delmas, E. , M. Besson , M. Brice , et al. 2019. “Analysing Ecological Networks of Species Interactions.” Biological Reviews 94, no. 1: 16–36. 10.1111/brv.12433. 29923657

[ece370389-bib-0020] Deregibus, D. , G. L. Campana , C. Neder , et al. 2023. “Potential Macroalgal Expansion and Blue Carbon Gains With Northern Antarctic Peninsula Glacial Retreat.” Marine Environmental Research 189: 106056. 10.1016/j.marenvres.2023.106056. 37385084

[ece370389-bib-0021] Deregibus, D. , M. L. Quartino , G. L. Campana , F. R. Momo , C. Wiencke , and K. Zacher . 2016. “Photosynthetic Light Requirements and Vertical Distribution of Macroalgae in Newly Ice‐Free Areas in Potter Cove, South Shetland Islands, Antarctica.” Polar Biology 39, no. 1: 153–166. 10.1007/s00300-015-1679-y.

[ece370389-bib-0022] Drossel, B. , A. McKane , and C. Quince . 2004. “The Impact of Nonlinear Functional Responses on the Long‐Term Evolution of Food Web Structure.” Journal of Theoretical Biology 229: 539–548. 10.1016/j.jtbi.2004.04.033. 15246789

[ece370389-bib-0023] Ducklow, H. , W. Fraser , M. Meredith , et al. 2013. “West Antarctic Peninsula: An Ice‐Dependent Coastal Marine Ecosystem in Transition.” Oceanography 26, no. 3: 190–203. 10.5670/oceanog.2013.62.

[ece370389-bib-0024] Dunne, J. A. , R. J. Williams , and N. D. Martinez . 2002. “Food‐Web Structure and Network Theory: The Role of Connectance and Size.” Proceedings of the National Academy of Sciences 99, no. 20: 12917–12922. 10.1073/pnas.192407699. PMC130560 12235364

[ece370389-bib-0025] Emmerson, M. , and J. M. Yearsley . 2004. “Weak Interactions, Omnivory and Emergent Food‐Web Properties.” Proceedings of the Royal Society of London. Series B: Biological Sciences 271, no. 1537: 397–405. 10.1098/rspb.2003.2592. PMC1691599 15101699

[ece370389-bib-0026] Garcia, M. D. , M. D. Fernández Severini , C. Spetter , et al. 2019. “Effects of Glacier Melting on the Planktonic Communities of Two Antarctic Coastal Areas (Potter Cove and Hope Bay) in Summer.” Regional Studies in Marine Science 30: 100731. 10.1016/j.rsma.2019.100731.

[ece370389-bib-0027] Gauzens, B. , A. Barnes , D. P. Giling , et al. 2019. “ *Fluxweb*: An r Package to Easily Estimate Energy Fluxes in Food Webs.” Methods in Ecology and Evolution 10, no. 2: 270–279. 10.1111/2041-210X.13109.

[ece370389-bib-0028] Gellner, G. , K. McCann , and A. Hastings . 2023. “Stable Diverse Food Webs Become More Common When Interactions Are More Biologically Constrained.” Proceedings of the National Academy of Sciences 120, no. 31: e2212061120. 10.1073/pnas.2212061120. PMC10400988 37487080

[ece370389-bib-0029] Gilarranz, L. J. , B. Rayfield , G. Liñán‐Cembrano , J. Bascompte , and A. Gonzalez . 2017. “Effects of Network Modularity on the Spread of Perturbation Impact in Experimental Metapopulations.” Science 357, no. 6347: 199–201. 10.1126/science.aal4122. 28706071

[ece370389-bib-0030] Gómez, I. , and P. Huovinen . 2020. Antarctic Seaweeds: Diversity, Adaptation and Ecosystem Services. Switzerland: Springer International Publishing. 10.1007/978-3-030-39448-6.

[ece370389-bib-0031] Grilli, J. , T. Rogers , and S. Allesina . 2016. “Modularity and Stability in Ecological Communities.” Nature Communications 7, no. 1: 12031. 10.1038/ncomms12031. PMC4931019 27337386

[ece370389-bib-0032] Guimerà, R. , and L. A. N. Amaral . 2005. “Cartography of Complex Networks: Modules and Universal Roles.” Journal of Statistical Mechanics: Theory and Experiment 2005, no. 2: P02001. 10.1088/1742-5468/2005/02/P02001. PMC2151742 18159217

[ece370389-bib-0033] Huang, Y. M. , M. O. Amsler , J. B. McClintock , C. D. Amsler , and B. J. Baker . 2007. “Patterns of Gammaridean Amphipod Abundance and Species Composition Associated With Dominant Subtidal Macroalgae From the Western Antarctic Peninsula.” Polar Biology 30, no. 11: 1417–1430. 10.1007/s00300-007-0303-1.

[ece370389-bib-0034] Hudson, L. N. , R. Emerson , G. B. Jenkins , et al. 2013. “Cheddar: Analysis and Visualisation of Ecological Communities in R.” Methods in Ecology and Evolution 4, no. 1: 99–104. 10.1111/2041-210X.12005.

[ece370389-bib-0035] Koenker, R. 2005. Quantile Regression. Cambridge: Cambridge University Press. 10.1017/CBO9780511754098.

[ece370389-bib-0036] Kortsch, S. , R. Frelat , L. Pecuchet , et al. 2021. “Disentangling Temporal Food Web Dynamics Facilitates Understanding of Ecosystem Functioning.” Journal of Animal Ecology 90, no. 5: 1205–1216. 10.1111/1365-2656.13447. 33608888

[ece370389-bib-0037] Kortsch, S. , R. Primicerio , M. Fossheim , A. V. Dolgov , and M. Aschan . 2015. “Climate Change Alters the Structure of Arctic Marine Food Webs due to Poleward Shifts of Boreal Generalists.” Proceedings of the Royal Society B: Biological Sciences 282, no. 1814: 20151546. 10.1098/rspb.2015.1546. PMC4571709 26336179

[ece370389-bib-0038] Landi, P. , H. O. Minoarivelo , Å. Brännström , C. Hui , and U. Dieckmann . 2018. “Complexity and Stability of Ecological Networks: A Review of the Theory.” Population Ecology 60, no. 4: 319–345. 10.1007/s10144-018-0628-3.

[ece370389-bib-0039] Marina, T. I. , V. Salinas , G. Cordone , et al. 2018. “The Food Web of Potter Cove (Antarctica): Complexity, Structure and Function.” Estuarine, Coastal and Shelf Science 200: 141–151. 10.1016/j.ecss.2017.10.015.

[ece370389-bib-0040] Marina, T. I. , L. A. Saravia , G. Cordone , V. Salinas , S. R. Doyle , and F. R. Momo . 2018. “Architecture of Marine Food Webs: To Be or Not Be a ‘Small‐World’.” PLoS One 13, no. 5: e0198217. 10.1371/journal.pone.0198217. 29813120 PMC5973612

[ece370389-bib-0041] Marina, T. I. , L. A. Saravia , and S. Kortsch . 2024. “New Insights Into the Weddell Sea Ecosystem Applying a Quantitative Network Approach.” Ocean Science 20, no. 1: 141–153. 10.5194/os-20-141-2024.

[ece370389-bib-0042] McCallum, H. 1999. Population Parameters: Estimation for Ecological Models, 1st ed. United States: Wiley. 10.1002/9780470757468.

[ece370389-bib-0043] McCann, K. , A. Hastings , and G. R. Huxel . 1998. “Weak Trophic Interactions and the Balance of Nature.” Nature 395, no. 6704: 794–798. 10.1038/27427.

[ece370389-bib-0044] Morlon, H. , S. Kefi , and N. D. Martinez . 2014. “Effects of Trophic Similarity on Community Composition.” Ecology Letters 17, no. 12: 1495–1506. 10.1111/ele.12356. 25292331

[ece370389-bib-0045] Neder, C. , V. Fofonova , A. Androsov , et al. 2022. “Modelling Suspended Particulate Matter Dynamics at an Antarctic Fjord Impacted by Glacier Melt.” Journal of Marine Systems 231: 103734. 10.1016/j.jmarsys.2022.103734.

[ece370389-bib-0046] Olivier, P. , and B. Planque . 2017. “Complexity and Structural Properties of Food Webs in the Barents Sea.” Oikos 126, no. 9: 1339–1346. 10.1111/oik.04138.

[ece370389-bib-0047] Pante, E. , and B. Simon‐Bouhet . 2013. “Marmap: A Package for Importing, Plotting and Analyzing Bathymetric and Topographic Data in R.” PLoS One 8, no. 9: e73051. 10.1371/journal.pone.0073051. 24019892 PMC3760912

[ece370389-bib-0048] Pawar, S. , A. I. Dell , and V. M. Savage . 2012. “Dimensionality of Consumer Search Space Drives Trophic Interaction Strengths.” Nature 486, no. 7404: 485–489. 10.1038/nature11131. 22722834

[ece370389-bib-0049] R Core Team . 2024. *R: The R Project for Statistical Computing* [Software]. https://www.r‐project.org/.

[ece370389-bib-0050] Rodriguez, I. D. , T. I. Marina , I. R. Schloss , and L. A. Saravia . 2022. “Marine Food Webs Are More Complex but Less Stable in Sub‐Antarctic (Beagle Channel, Argentina) Than in Antarctic (Potter Cove, Antarctic Peninsula) Regions.” Marine Environmental Research 174: 105561. 10.1016/j.marenvres.2022.105561. 35026725

[ece370389-bib-0051] Rückamp, M. , M. Braun , S. Suckro , and N. Blindow . 2011. “Observed Glacial Changes on the King George Island Ice Cap, Antarctica, in the Last Decade.” Global and Planetary Change 79, no. 1–2: 99–109. 10.1016/j.gloplacha.2011.06.009.

[ece370389-bib-0052] Sahade, R. , C. Lagger , L. Torre , et al. 2015. “Climate Change and Glacier Retreat Drive Shifts in an Antarctic Benthic Ecosystem.” Science Advances 1, no. 10: e1500050. 10.1126/sciadv.1500050. 26702429 PMC4681327

[ece370389-bib-0053] Salinas, V. , G. Cordone , T. I. Marina , and F. R. Momo . 2024. “Estimating the Impact of Biodiversity Loss in a Marine Antarctic Food Web.” Diversity 16, no. 1: 63. 10.3390/d16010063.

[ece370389-bib-0054] Saravia, L. A. 2024. *Multiweb: Ecological Network Analyses Including Multiplex Networks* [Software]. https://github.com/lsaravia/multiweb

[ece370389-bib-0055] Saravia, L. A. , T. I. Marina , N. P. Kristensen , M. De Troch , and F. R. Momo . 2022. “Ecological Network Assembly: How the Regional Metaweb Influences Local Food Webs.” Journal of Animal Ecology 91, no. 3: 630–642. 10.1111/1365-2656.13652. 34951015

[ece370389-bib-0056] Schloss, I. R. , D. Abele , S. Moreau , et al. 2012. “Response of Phytoplankton Dynamics to 19‐Year (1991–2009) Climate Trends in Potter Cove (Antarctica).” Journal of Marine Systems 92, no. 1: 53–66. 10.1016/j.jmarsys.2011.10.006.

[ece370389-bib-0057] Schloss, I. R. , G. A. Ferreyra , and D. Ruiz‐Pino . 2002. “Phytoplankton Biomass in Antarctic Shelf Zones: A Conceptual Model Based on Potter Cove, King George Island.” Journal of Marine Systems 36, no. 3–4: 129–143. 10.1016/S0924-7963(02)00183-5.

[ece370389-bib-0058] Stouffer, D. B. , and J. Bascompte . 2011. “Compartmentalization Increases Food‐Web Persistence.” Proceedings of the National Academy of Sciences 108, no. 9: 3648–3652. 10.1073/pnas.1014353108. PMC3048152 21307311

[ece370389-bib-0059] Turner, J. , N. E. Barrand , T. J. Bracegirdle , et al. 2014. “Antarctic Climate Change and the Environment: An Update.” Polar Record 50, no. 3: 237–259. 10.1017/S0032247413000296.

[ece370389-bib-0060] Wootton, J. T. , and M. Emmerson . 2005. “Measurement of Interaction Strength in Nature.” Annual Review of Ecology, Evolution, and Systematics 36, no. 1: 419–444. 10.1146/annurev.ecolsys.36.091704.175535.

[ece370389-bib-0061] Wootton, K. L. 2017. “Omnivory and Stability in Freshwater Habitats: Does Theory Match Reality?” Freshwater Biology 62, no. 5: 821–832. 10.1111/fwb.12908.

