# Supplementary material for: Potter Cove's Heavyweights: Estimation of Species' Interaction Strength of an Antarctic Food Web
Source: Ecol Evol. 2024 Nov 3;14(11):e70389. doi: 10.1002/ece3.70389 (PMC11531876; doi:10.1002/ece3.70389)
Supplement: Supplementary file 1 — Data S1. [file ECE3-14-e70389-s001.pdf]

Supporting Information for *Potter Cove's Heavyweights: Estimation of species' interaction strength of an Antarctic food web*

Iara Diamela Rodriguez and Leonardo Ariel Saravia

Materials and methods

I. Equations for calculating species properties

<u>Interaction strength</u>

We used the estimation of the interaction strength as a weighted property for the species of the Potter Cove food web. The main equation we used to estimate the interaction strength (IS) was:

$$IS = \frac{\alpha\, x_R\, m_R}{m_C}$$

where α is the search rate, x_R is the resource density and m_R and m_C the body mass for the resource and the consumer, respectively (Pawar et al., 2012). We assume the case where resources are scarce because this resembles field conditions (figure 3.e and f and equation 3 from Pawar et al. (2012). Then the search rate for 2D interactions is calculated as:

$$\alpha = \alpha_{2D} m_C^{0.68 \pm 0.12}$$

For 3D interactions it is calculated as:

$$\alpha = \alpha_{3D} m_C^{1.05 \pm 0.08}$$

where $\alpha_{2D} = 10^{-3.08}$ and $\alpha_{3D} = 10^{-1.77}$ are the intercepts for each interaction dimensionality.

When empirical resource density (x_R) was not available, we estimated it according to the equation S18 and supplementary figures 2.i and j (individuals/m2 - m3) from Pawar et al. (2012):

$$x_R = x_0 m_R^{-p_x}$$

where p_x is -0.79 ± 0.08 for 2D and -0.86 ± 0.07 for 3D.

Topological properties

As unweighted properties we calculated species trophic level, degree, omnivory and trophic similarity.

The **trophic level** was calculated for every species based on its position in the food web using the ‘‘prey-averaged technique’’. This metric is based on the trophic position of all prey in the diet of a consumer, where primary producers are assigned a TL = 0, and assumes that all links are quantitatively equivalent (each taxon eats the same amount of every prey). TP is calculated for taxon i from the binary matrix of trophic interactions as:

$$TP_i = \frac{\sum_j TP_j}{n_i} + 1$$

where n_i is the total number of prey taxa consumed by taxon i, and TP_j represents the trophic position of all prey items (j) of taxon i (Thompson et al., 2007)

The **degree** k is simply the total number of feeding links in which the species participates. It was calculated as:

$$L = \sum_{i=1}^{S} k_i$$

where L is the total number of feeding links for the ith species in the food web; here denoted as k_i.

Omnivory is calculated as the variance of the trophic levels of a consumer's preys. For species i:

$$OI = \sum_{j=1}^{n} (TL_j - TL)^2\, DC_{ij}$$

where n is the number of species in the system, TL_j is the trophic level of prey j, TL is the average trophic level of the preys, and DC_{ij} is the fraction of prey (j) in the average diet of predator (i) (Libralato, 2008).

The **trophic similarity** (TS) between every pair of species in the food web was calculated using the following algorithm:

$$TS = \frac{c}{a + b + c}$$

where c is the number of predators and prey common to the two species, a is the number of predators and prey unique to one species, and b is the number of predators and prey unique to the other species. When the two species have the same set of predators and

prey, $TS = 1$; when the two species have no common predators or common prey, $TS = 0$ (Martinez, 1991).

The **intermodule connectivity** (IMC) estimates the links distribution of species *i* among modules, as follows:

$$IMC_i = 1 - \sum_s \frac{k_{is}}{k_i}$$

where k_i is the total number of links of species *i* and k_{is} is the number of links of species *i* to species in module *s*.

Results

I. Interaction strength distribution

Table S1. Model comparison for the distribution of interaction strengths of the Potter Cove food web. Order by best fit. References: AIC = Akaike Information Criterion, △AIC = difference with best fit. *Indicates best-fit model.

Model	AIC	△AIC
Gamma*	-9703.21	0
Power-law	-9645.35	57.85
Log-Normal	-9431.38	271.82
Exponential	-5654.65	4048.56
Normal	-4095.24	5607.96
Uniform	-3251.24	6451.97

II. Species interaction strength and topological properties

Table S2. Results of quantile regression analyses, coefficients and significance levels for log total interaction strength and topological properties (trophic level, degree, omnivory, and trophic similarity) at quantiles 0.25, 0.5, and 0.75. * Indicates statistical significance (p-value <0.05).

		Trophic level	Degree	Omnivory	Trophic similarity
q25	Coefficient value	0.228	0.970	-1.865	13.974
	p-value	0.680	0.000*	0.588	0.243
q50	Coefficient value	-0.143	0.864	-0.145	12.756
	p-value	0.648	0.009*	0.942	0.002*
q75	Coefficient value	-0.749	0.822	-3.885	11.535
	p-value	0.001*	0.007*	0.018*	0.109*

III. Species impact on food web stability and fragmentation

Table S3. Effect of species extinction by decreasing (a) interaction strength (weighted property), (b) trophic level, (c) degree, (d) omnivory and (e) trophic similarity (unweighted properties) on the Potter Cove food web stability and fragmentation. Stability = mean maximum eigenvalue, Q1 = first quartile of the stability distribution, Q3 = third quartile of the stability distribution, Size = food web size. On darker green species that when deleted have great impact on food web stability. On lighter green species that when removed fragmented the food web.

Last deleted sp.	Stability	Q1	Q3	Size	Components	Proportion of deleted sp.
**(a) EXTINTIONS BY DECREASING INTERACTION STRENGTH**						
Total	0,029	0,019	0,045	110	1	0,00
Fresh detritus	0,029	0,018	0,043	109	1	0,01
Aged detritus	0,030	0,020	0,046	108	1	0,02
Prostebbingia sp.	**0,012**	**0,005**	**0,024**	**107**	**1**	**0,03**
Prostebbingia gracilis	**0,005**	**0,003**	**0,009**	**106**	**1**	**0,04**
Necromass	0,004	0,003	0,009	105	1	0,05
Gondogeneia antarctica	0,003	0,002	0,004	104	1	0,05
Palmaria decipiens	0,003	0,002	0,004	103	1	0,06
Laevilacunaria antarctica	0,003	0,002	0,004	102	1	0,07
Gammaridea	0,003	0,002	0,004	101	1	0,08
Polychaeta	0,003	0,002	0,004	100	1	0,09
Desmarestia menziesii	0,003	0,002	0,004	99	1	0,10
Desmarestia anceps	0,003	0,002	0,004	98	1	0,11
Desmarestia antarctica	0,003	0,002	0,004	97	1	0,12
Gigartina skottsbergii	0,003	0,002	0,004	96	1	0,13
Iridaea cordata	0,003	0,002	0,004	95	1	0,14
Plocamium cartilagineum	0,003	0,002	0,004	94	1	0,15
Cheirimedon femoratus	0,003	0,002	0,004	93	1	0,15
Porifera	0,003	0,002	0,004	92	1	0,16
Myriogramme manginii	0,003	0,002	0,004	91	1	0,17
Pariphimedia integricauda	0,003	0,002	0,004	90	1	0,18
Paradexamine fissicauda	**0,003**	**0,002**	**0,004**	**89**	**2**	**0,19**
Oligochaeta	0,003	0,002	0,004	88	2	0,20
Oradarea bidentata	0,003	0,002	0,004	87	2	0,21
Orchomenella sp.	0,003	0,002	0,004	86	2	0,22
Eatoniella sp.	0,003	0,002	0,004	85	2	0,23
Ophionotus victoriae	0,002	0,001	0,003	84	2	0,24
Priapulida	0,002	0,001	0,003	83	2	0,25
Eurymera monticulosa	0,002	0,002	0,003	82	2	0,25
Notothenia coriiceps	**0,002**	**0,001**	**0,003**	**81**	**14**	**0,26**
Odontaster validus	0,002	0,001	0,003	80	14	0,27
Adenocystis utricularis	0,002	0,001	0,003	79	13	0,28
Polynoidae	0,002	0,001	0,003	78	13	0,29
Hydrozoa	0,002	0,001	0,003	77	13	0,30
Charcotia obesa	0,002	0,001	0,003	76	13	0,31
Cumacea	0,002	0,001	0,003	75	13	0,32
Stylo_Myca	0,001	0,001	0,002	74	14	0,33
Aequiyoldia eightsii	0,001	0,001	0,002	73	14	0,34
Hippomedon kergueleni	0,001	0,001	0,002	72	14	0,35

Polyplacophora	0,001	0,001	0,002	71	14	0,35
Laternula elliptica	0,001	0,001	0,002	70	14	0,36
Notothenia rossii	0,001	0,001	0,002	69	15	0,37
Tanaidacea	0,001	0,001	0,002	68	15	0,38
Phaeurus antarcticus	0,001	0,001	0,003	67	14	0,39
Cephalopoda	0,002	0,001	0,003	66	14	0,40
Nereididae	0,001	0,001	0,003	65	14	0,41
Serolis sp.	0,001	0,001	0,002	64	14	0,42
Georgiella confluens	0,002	0,001	0,003	63	13	0,43
Curdiea racovitzae	0,001	0,001	0,002	62	12	0,44
Monostroma hariotii	0,002	0,001	0,003	61	11	0,45
Mysida	0,001	0,001	0,002	60	11	0,45
**(b) EXTINTIONS BY DECREASING TROPHIC LEVEL**						
Total	0,031	0,019	0,046	110	1	0,00
Chaenocephalus aceratus	0,028	0,018	0,044	109	1	0,01
Urticinopsis antarctica	0,029	0,018	0,044	108	1	0,02
Parachaenichthys charcoti	0,030	0,018	0,045	107	1	0,03
Octopoda	0,031	0,018	0,046	106	1	0,04
Trematomus bernacchii	0,029	0,018	0,043	105	1	0,05
Lindbergichthys nudifrons	0,028	0,018	0,044	104	1	0,05
Cephalopoda	0,030	0,019	0,046	103	1	0,06
Hyperiidea	0,030	0,020	0,044	102	1	0,07
Harpagifer antarcticus	0,030	0,019	0,044	101	1	0,08
Trematomus newnesi	0,031	0,019	0,047	100	1	0,09
Barrukia cristata	0,030	0,019	0,043	99	1	0,10
Glyptonotus antarcticus	0,029	0,018	0,045	98	1	0,11
Gobionotothen gibberifrons	0,030	0,018	0,045	97	1	0,12
Ophionotus victoriae	0,030	0,019	0,047	96	1	0,13
Priapulida	0,030	0,018	0,044	95	1	0,14
Euphausia superba	0,029	0,018	0,044	94	1	0,15
Hydrozoa	0,028	0,018	0,043	93	1	0,15
Polynoidae	0,030	0,019	0,047	92	1	0,16
Parborlasia corrugatus	0,029	0,018	0,044	91	1	0,17
Notothenia rossii	0,030	0,019	0,045	90	1	0,18
Nemertea	0,030	0,019	0,044	89	1	0,19
Salpidae	0,030	0,019	0,045	88	1	0,20
Odontaster meridionalis	0,030	0,018	0,045	87	1	0,21
Odontaster validus	0,031	0,018	0,046	86	1	0,22
Perknaster aurorae	0,028	0,018	0,043	85	1	0,23
Serolis sp.	0,029	0,018	0,044	84	1	0,24
Notothenia coriiceps	**0,029**	**0,019**	**0,044**	**83**	**9**	**0,25**
Polychaeta	0,029	0,019	0,045	82	9	0,25
Diplasterias brucei	0,029	0,018	0,044	81	9	0,26
Sterechinus neumayeri	0,030	0,018	0,046	80	9	0,27
Aglaophamus trissophyllus	0,031	0,019	0,044	79	9	0,28
Bovallia gigantea	0,030	0,020	0,045	78	9	0,29
Gastropoda	0,031	0,019	0,046	77	9	0,30
Doris kerguelenensis	0,030	0,019	0,045	76	9	0,31
Perknaster fuscus antarticus	0,031	0,020	0,046	75	9	0,32
Nereididae	0,031	0,019	0,045	74	9	0,33
Hemiarthrum setulosum	0,031	0,020	0,047	73	9	0,34
Mysida	0,031	0,019	0,047	72	9	0,35
Malacobelemnon daytoni	0,028	0,018	0,044	71	9	0,35
Neobuccinum eatoni	0,028	0,018	0,045	70	9	0,36
Cheirimedon femoratus	0,029	0,018	0,044	69	9	0,37
Orchomenella sp.	0,030	0,018	0,045	68	9	0,38

Aequiyoldia eightsii	0,029	0,018	0,044	67	9	0,39
Copepoda	0,030	0,019	0,046	66	9	0,40
Polyplacophora	0,029	0,018	0,045	65	9	0,41
Eatoniella sp.	0,030	0,019	0,046	64	9	0,42
Gammaridea	0,026	0,015	0,045	63	9	0,43
Ascidiacea	0,028	0,016	0,045	62	9	0,44
Terebellidae	0,027	0,015	0,043	61	9	0,45
Ostracoda	0,028	0,016	0,047	60	9	0,45
**(c) EXTINTIONS BY DECREASING DEGREE**						
Total	0,029	0,018	0,044	110	1	0,00
Notothenia coriiceps	**0,028**	**0,019**	**0,044**	**109**	**9**	**0,01**
Fresh detritus	0,030	0,019	0,046	108	9	0,02
Notothenia rossii	0,029	0,018	0,045	107	9	0,03
Trematomus bernacchii	0,029	0,018	0,043	106	9	0,04
Benthic Diatomea	0,030	0,018	0,043	105	9	0,05
Ophionotus victoriae	0,029	0,018	0,044	104	9	0,05
Polychaeta	0,029	0,017	0,044	103	9	0,06
Copepoda	0,030	0,018	0,046	102	9	0,07
Harpagifer antarcticus	0,030	0,018	0,044	101	9	0,08
Aged detritus	0,030	0,019	0,046	100	9	0,09
Trematomus newnesi	0,030	0,018	0,044	99	10	0,10
Necromass	0,031	0,019	0,046	98	10	0,11
Odontaster validus	0,031	0,019	0,045	97	10	0,12
Phytoplankton	0,030	0,019	0,045	96	11	0,13
Nereididae	0,031	0,019	0,045	95	11	0,14
Gammaridea	0,027	0,013	0,046	94	11	0,15
Lindbergichthys nudifrons	0,027	0,013	0,046	93	12	0,15
Sterechinus neumayeri	0,028	0,015	0,046	92	12	0,16
Gondogeneia antarctica	**0,019**	**0,003**	**0,040**	**91**	**14**	**0,17**
Ostracoda	0,016	0,002	0,040	90	14	0,18
Bovallia gigantea	0,016	0,003	0,038	89	16	0,19
Zooplankton	0,016	0,003	0,040	88	18	0,20
Hydrozoa	0,016	0,002	0,040	87	18	0,21
Polynoidae	0,016	0,003	0,041	86	19	0,22
Gastropoda	0,016	0,003	0,040	85	20	0,23
Porifera	0,018	0,003	0,040	84	23	0,24
Prostebbingia gracilis	**0,002**	**0,001**	**0,003**	**83**	**23**	**0,25**
Prostebbingia sp.	0,001	0,001	0,002	82	26	0,25
Euphausia superba	0,001	0,001	0,002	81	27	0,26
Mysida	0,001	0,001	0,002	80	28	0,27
Gobionotothen gibberifrons	0,001	0,001	0,002	79	30	0,28
Glyptonotus antarcticus	0,001	0,001	0,002	78	30	0,29
Nemertea	0,001	0,001	0,002	77	32	0,30
Salpidae	0,001	0,001	0,002	76	33	0,31
Parborlasia corrugatus	0,001	0,000	0,001	75	34	0,32
Aequiyoldia eightsii	0,000	0,000	0,001	74	34	0,33
Stylo_Myca	0,000	0,000	0,001	73	35	0,34
Bryozoa	0,000	0,000	0,001	72	35	0,35
Palmaria decipiens	0,000	0,000	0,001	71	37	0,35
Laevilacunaria antarctica	0,000	0,000	0,001	70	45	0,36
Orchomenella sp.	0,000	0,000	0,001	69	44	0,37
Polyplacophora	0,000	0,000	0,001	68	45	0,38
Nacella concinna	0,000	0,000	0,001	67	47	0,39
Paradexamine fissicauda	0,000	0,000	0,001	66	48	0,40
Hyperiidea	0,000	0,000	0,001	65	47	0,41
Urticinopsis antarctica	0,000	0,000	0,001	64	48	0,42

Charcotia obesa	0,000	0,000	0,001	63	47	0,43
Neobuccinum eatoni	0,000	0,000	0,001	62	49	0,44
Rosella antartica	0,000	0,000	0,000	61	50	0,45
Tanaidacea	0,000	0,000	0,000	60	49	0,45
**(d) EXTINTIONS BY DECREASING OMNIVORY**						
Total	0,031	0,019	0,046	110	1	0,00
Zooplankton	0,029	0,018	0,045	109	1	0,01
Hydrozoa	0,028	0,017	0,044	108	1	0,02
Diplasterias brucei	0,030	0,018	0,046	107	1	0,03
Nemertea	0,029	0,018	0,045	106	1	0,04
Aglaophamus trissophyllus	0,030	0,019	0,045	105	1	0,05
Gobionotothen gibberifrons	0,028	0,017	0,043	104	1	0,05
Glyptonotus antarcticus	0,029	0,018	0,045	103	1	0,06
Odontaster validus	0,030	0,019	0,045	102	1	0,07
Aequiyoldia eightsii	0,029	0,018	0,043	101	1	0,08
Polynoidae	0,028	0,018	0,044	100	1	0,09
Gastropoda	0,029	0,019	0,046	99	1	0,10
Polyplacophora	0,028	0,017	0,044	98	1	0,11
Serolis sp.	0,029	0,019	0,044	97	1	0,12
Ophionotus victoriae	0,028	0,018	0,044	96	1	0,13
Neobuccinum eatoni	0,029	0,018	0,046	95	1	0,14
Charcotia obesa	0,029	0,019	0,043	94	1	0,15
Sterechinus neumayeri	0,029	0,018	0,046	93	1	0,15
Barrukia cristata	0,030	0,019	0,045	92	1	0,16
Parachaenichthys charcoti	0,028	0,018	0,045	91	1	0,17
Orchomenella sp.	0,029	0,018	0,043	90	1	0,18
Perknaster fuscus antarticus	0,031	0,019	0,045	89	1	0,19
Chaenocephalus aceratus	0,029	0,019	0,045	88	1	0,20
Hyperiidea	0,029	0,018	0,043	87	1	0,21
Doris kerguelenensis	0,029	0,018	0,043	86	1	0,22
Trematomus bernacchii	0,029	0,019	0,045	85	1	0,23
Notothenia rossii	0,030	0,018	0,045	84	1	0,24
Notothenia coriiceps	**0,029**	**0,018**	**0,045**	**83**	**9**	**0,25**
Parborlasia corrugatus	0,030	0,018	0,044	82	9	0,25
Eatoniella sp.	0,030	0,018	0,045	81	9	0,26
Trematomus newnesi	0,030	0,019	0,045	80	9	0,27
Nereididae	0,031	0,020	0,046	79	9	0,28
Harpagifer antarcticus	0,030	0,019	0,044	78	9	0,29
Urticinopsis antarctica	0,029	0,019	0,044	77	9	0,30
Euphausia superba	0,030	0,018	0,044	76	9	0,31
Bovallia gigantea	0,029	0,019	0,044	75	9	0,32
Polychaeta	0,030	0,018	0,045	74	9	0,33
Octopoda	0,031	0,019	0,047	73	9	0,34
Lindbergichthys nudifrons	0,030	0,020	0,044	72	9	0,35
Salpidae	0,031	0,019	0,045	71	9	0,35
Cheirimedon femoratus	0,031	0,018	0,046	70	9	0,36
Odontaster meridionalis	0,029	0,018	0,045	69	9	0,37
Aged detritus	0,031	0,020	0,046	68	9	0,38
Benthic Diatomea	0,030	0,018	0,046	67	12	0,39
Fresh detritus	0,031	0,018	0,045	66	19	0,40
Phytoplankton	0,031	0,018	0,046	65	25	0,41
Copepoda	0,030	0,018	0,046	64	26	0,42
Porifera	0,030	0,018	0,045	63	26	0,43
Eurymera monticulosa	0,030	0,018	0,044	62	26	0,44
Gitanopsis squamosa	0,031	0,019	0,046	61	26	0,45
Gondogeneia antarctica	0,024	0,010	0,043	60	27	0,45

(e) EXTINTIONS BY DECREASING TROPHIC SIMILARITY						
Total	0,029	0,018	0,044	110	1	0,00
Orchomenella sp.	0,029	0,018	0,044	109	1	0,01
Djerboa furcipes	0,029	0,018	0,045	108	1	0,02
Ascidiacea	0,029	0,019	0,045	107	1	0,03
Ostracoda	0,029	0,018	0,045	106	1	0,04
Aequiyoldia eightsii	0,029	0,017	0,046	105	1	0,05
Cheirimedon femoratus	0,031	0,018	0,047	104	1	0,05
Tanaidacea	0,028	0,018	0,044	103	1	0,06
Hydrozoa	0,029	0,018	0,045	102	1	0,07
Charcotia obesa	0,030	0,019	0,045	101	1	0,08
Mysida	0,030	0,019	0,045	100	1	0,09
Gitanopsis squamosa	0,031	0,018	0,046	99	1	0,10
Eurymera monticulosa	0,032	0,019	0,047	98	1	0,11
Pariphimedia integricauda	0,030	0,018	0,046	97	1	0,12
Probolisca ovata	0,029	0,017	0,044	96	1	0,13
Nereididae	0,029	0,018	0,045	95	1	0,14
Oradarea bidentata	0,032	0,017	0,048	94	1	0,15
Margarella antarctica	0,030	0,017	0,046	93	1	0,15
Copepoda	0,029	0,016	0,045	92	1	0,16
Bovallia gigantea	0,027	0,016	0,044	91	1	0,17
Glyptonotus antarcticus	0,030	0,017	0,047	90	1	0,18
Gastropoda	0,031	0,017	0,048	89	1	0,19
Salpidae	0,030	0,017	0,047	88	1	0,20
Gammaridea	0,025	0,012	0,045	87	1	0,21
Laternula elliptica	0,027	0,012	0,047	86	1	0,22
Neuroglossum delesseriae	0,026	0,012	0,045	85	1	0,23
Trematocarpus antarcticus	0,025	0,011	0,045	84	1	0,24
Urospora penicilliformis	0,026	0,012	0,046	83	1	0,25
Hyperiidea	0,026	0,012	0,044	82	1	0,25
Hippomedon kergueleni	0,027	0,013	0,046	81	1	0,26
Euphausia superba	0,025	0,012	0,044	80	1	0,27
Iridaea cordata	0,026	0,014	0,045	79	1	0,28
Plocamium cartilagineum	0,023	0,010	0,040	78	1	0,29
Polychaeta	0,022	0,009	0,041	77	1	0,30
Desmarestia menziesii	0,020	0,009	0,038	76	1	0,31
Nacella concinna	**0,021**	**0,009**	**0,038**	**75**	**2**	**0,32**
Desmarestia anceps	0,017	0,007	0,032	74	2	0,33
Desmarestia antarctica	0,013	0,006	0,025	73	2	0,34
Paradexamine fissicauda	0,014	0,006	0,029	72	3	0,35
Aglaophamus trissophyllus	0,015	0,006	0,030	71	3	0,35
Gigartina skottsbergii	0,008	0,004	0,019	70	3	0,36
Bryozoa	0,010	0,005	0,023	69	3	0,37
Polyplacophora	0,008	0,004	0,020	68	3	0,38
Ophionotus victoriae	0,009	0,004	0,021	67	3	0,39
Nemertea	0,009	0,003	0,021	66	3	0,40
Polynoidae	0,007	0,003	0,018	65	3	0,41
Cumacea	0,009	0,004	0,021	64	3	0,42
Pseudorchomene plebs	0,008	0,003	0,020	63	3	0,43
Prostebbingia gracilis	0,003	0,002	0,004	62	3	0,44
Sterechinus neumayeri	0,003	0,002	0,004	61	3	0,45
Curdiea racovitzae	0,003	0,002	0,004	60	3	0,45

EXTINTIONS BY DECREASING INTERMODULE CONNECTIVITY						
Total	0,030	0,019	0,044	110	1	0,00
Glyptonotus antarcticus	0,030	0,020	0,045	109	1	0,01

Necromass	0,029	0,018	0,045	108	1	0,02
Orchomenella sp,	0,029	0,017	0,044	107	1	0,03
Djerboa furcipes	0,029	0,018	0,044	106	1	0,04
Harpagifer antarcticus	0,029	0,018	0,045	105	1	0,05
Trematomus newnesi	0,028	0,018	0,044	104	1	0,05
Pseudorchomene plebs	0,028	0,018	0,043	103	1	0,06
Gammaridea	0,025	0,014	0,043	102	1	0,07
Gobionotothen gibberifrons	0,028	0,014	0,045	101	1	0,08
Trematomus bernacchii	0,027	0,014	0,044	100	1	0,09
Aged detritus	0,027	0,014	0,045	99	1	0,10
Fresh detritus	0,026	0,014	0,043	98	1	0,11
Polychaeta	0,029	0,014	0,047	97	1	0,12
Gitanopsis squamosa	0,028	0,014	0,045	96	1	0,13
Copepoda	0,026	0,014	0,043	95	1	0,14
Phytoplankton	0,027	0,014	0,045	94	1	0,15
Cheirimedon femoratus	0,027	0,014	0,047	93	1	0,15
Octopoda	0,026	0,014	0,044	92	1	0,16
Aequiyoldia eightsii	0,028	0,013	0,045	91	1	0,17
Ostracoda	0,027	0,014	0,045	90	1	0,18
Ascidiacea	0,026	0,013	0,044	89	1	0,19
Hydrozoa	0,026	0,014	0,044	88	1	0,20
Hippomedon kergueleni	0,027	0,013	0,045	87	1	0,21
Notothenia coriiceps	**0,028**	**0,015**	**0,046**	**86**	**9**	**0,22**
Aglaophamus trissophyllus	0,028	0,014	0,046	85	9	0,23
Mysida	0,026	0,014	0,044	84	9	0,24
Cumacea	0,027	0,014	0,044	83	9	0,25
Nereididae	0,027	0,014	0,044	82	9	0,25
Lindbergichthys nudifrons	0,026	0,013	0,045	81	9	0,26
Bovallia gigantea	0,028	0,014	0,046	80	9	0,27
Paradexamine fissicauda	0,027	0,013	0,044	79	10	0,28
Gastropoda	0,027	0,013	0,046	78	10	0,29
Notothenia rossii	0,027	0,014	0,044	77	11	0,30
Tanaidacea	0,027	0,013	0,044	76	11	0,31
Benthic Diatomea	0,027	0,014	0,043	75	12	0,32
Serolis sp.	0,026	0,013	0,046	74	12	0,33
Margarella antarctica	0,026	0,014	0,046	73	11	0,34
Charcotia obesa	0,026	0,014	0,044	72	11	0,35
Ophionotus victoriae	0,026	0,014	0,043	71	11	0,35
Probolisca ovata	0,026	0,013	0,044	70	10	0,36
Polynoidae	0,026	0,013	0,043	69	10	0,37
Oradarea bidentata	0,026	0,013	0,045	68	10	0,38
Barrukia cristata	0,027	0,014	0,045	67	10	0,39
Polyplacophora	0,028	0,014	0,044	66	10	0,40
Sterechinus neumayeri	0,027	0,014	0,044	65	11	0,41
Hemiarthrum setulosum	0,027	0,014	0,045	64	12	0,42
Nacella concinna	0,029	0,014	0,046	63	14	0,43
Priapulida	0,026	0,013	0,045	62	14	0,44
Eurymera monticulosa	0,027	0,014	0,046	61	14	0,45
Laternula elliptica	0,026	0,013	0,045	60	14	0,45

Referencias

Libralato, S. (2008). System Omnivory Index. En S. E. Jørgensen & B. D. Fath (Eds.), *Encyclopedia of Ecology* (pp. 3472-3477). Academic Press. https://doi.org/10.1016/B978-008045405-4.00135-X

Martinez, N. D. (1991). Artifacts or Attributes? Effects of Resolution on the Little Rock Lake Food Web. *Ecological Monographs*, *61*(4), 367-392. https://doi.org/10.2307/2937047

Pawar, S., Dell, A. I., & Van M. Savage. (2012). Dimensionality of consumer search space drives trophic interaction strengths. *Nature*, *486*(7404), 485-489. https://doi.org/10.1038/nature11131

Thompson, R. M., Hemberg, M., Starzomski, B. M., & Shurin, J. B. (2007). TROPHIC LEVELS AND TROPHIC TANGLES: THE PREVALENCE OF OMNIVORY IN REAL FOOD WEBS. *Ecology*, *88*(3), 612-617. https://doi.org/10.1890/05-1454
